# Backward sensing FUS modulated light from a brain phantom through human adult skull specimen

**DOI:** 10.1364/BOE.606753

**Published:** 2026-08-24

**Authors:** Zuomin Zhao, Mohammadreza Omidali, Triana Rahayu, Teemu Myllylä

**Affiliations:** 1 Optoelectronics and Measurement Techniques Unit, Advanced Electronics Centre (AEC), Faculty of Information Technology and Electrical Engineering, University of Oulu, 90014 Oulu, Finland; 2 Research Unit of Health Sciences and Technology, Faculty of Medicine, University of Oulu, 90014 Oulu, Finland Infotech Institute, University of Oulu, Finland

## Abstract

Near-infrared (NIR) diffusive reflection spectroscopy is widely useful in medical applications. However, its spatial resolution is low, especially when studying deep tissue. Focused ultrasound (FUS) modulated light, also known as acousto-optic (AO) can be used to improve the spatial resolution of optical sensing of deep tissue based on detection of the modulated photons from focal area of the FUS. In this study, we investigated FUS-modulated light in deep bio-tissue phantoms in reflection mode through a human adult skull specimen. The experiment setup used an optical fiber (source fiber) to guide coherent light illuminating the sample surface, and another optical fiber (detection fiber) to receive optical signal to be sent to a photoreceiver. First, we investigated AO signal coming from deep tissue, accepted by the detection fiber located 3 cm away from the source fiber. Then we studied the effects of ultrasonic modulation frequency and detection fiber diameter on the AO signal intensity. Finally, we studied the effect of adult human skull specimen on AO signal from brain phantom which was in the skull. The results show that it is possible to detect AO signals from a brain phantom through a human skull, although the skull specimen reduced the AO signal intensity by approximately 50% at the applied FUS pressure level. This study provides useful insights toward future AO sensing of the human brain when using FUS-modulated light.

## 1. Introduction

Optical methods and optical imaging have become widely used in medical research and applications because light is non-ionized radiation and tissue components have good optical contrast. However, light beam has a quite limit ability for penetrating human skin (penetration depth 1–3 mm in visible to NIR wavelengths [1,2]), impeding it for deep tissue imaging and sensing. On the other hand, light can diffuse through skin, reaching internal tissues at several centimeters’ depth by multiple optical scattering [3,4]. Detecting these photons coming from deep tissues makes the development of diffuse optical imaging such as optical tomography (OT) and functional near infrared spectroscopy (fNIRS). However, diffuse optical imaging has low spatial resolution (in millimeters’ order [5]) because of fast and multiple scattering pathlengths of photons. This drawback has been greatly improved by photoacoustic imaging or tomography (PAI/PAT) [6,7] and acousto-optic (AO) imaging or tomography [8–11] since 1990s. Both are hybrid techniques and utilize the high resolution of ultrasound signal where PAI detects laser-generated ultrasonic waves at optical absorbing components and AO detects US-modulated optical signal from US region in tissue.

The mechanisms of US modulation of light, from the point view of US disturbing the optical filed in the scattering media, can be divided into two groups [12]. One group is based on US-induced variations of the optical properties of the media. When US wave propagates in a scattering medium, the medium is compressed and rarified depending on location and time, resulting in variations of scatterers’ shape, location, and density distribution. These cause medium’s optical properties such as absorption coefficient, scattering coefficient, and index of refraction to vary. These varying mechanisms weakly modulate incoherent light which have been difficult to observe experimentally [13,14]. The second group is based on the US-induced variation of the optical phase of a coherent light. The phase variation is produced by two mechanisms: the first is US-induced displacement of scatterers which accumulates modulated lengths along light paths, and the second is US-modulated index of refraction of the media which modulates the optical phase among the consecutive scattering events. Both mechanisms require a coherent light to have sufficient coherent length. The relative influence of both mechanisms depend on properties of used light, US, and media, for example, the US-modulated index of refraction becomes dominant when the acoustic wavelength becomes shorter than a critical fraction of the mean free path of the photons (∼ 0.559 × optical scattering coefficient) [15,16].

On the other hand, from the point view of two waves mixing, US-modulated light can be explained by mixing US and optical frequencies, in which US generates sidebands on light frequency to be shifted by an integral multiple of US frequency (called as US “tagged” photons). Usually, ±1^st^ order sidebands contain a significant number of tagged photons [9,17], and only one sideband is detected for sensing or imaging in practice. Based on Monte Carlo simulation, the modulation depth at ∼1 MHz US frequency is in the order of 10^−5–^10^−3^ in a typical tissue at the order of centimeter depth [18]. Moreover, it has a close relationship between modulation depth and the contrast of the speckle patterns produced by US-induced optical phase of a coherent light.

The benefit of AO detection is that besides optical absorption it also senses optical scattering related components. Moreover, it improves spatial resolution in optical sensing and imaging, by using US to tag diffused photons or modulate light in US area. These tagged or modulated photons at the US frequency have spatial information of the focus area. Hence, the spatial resolution is dependent on the spatial accuracy of the US focusing.

Optical and ultrasonic brain sensing and imaging have been one of hottest research area because they are convenient, low cost, and non-ionized radiation tools for studying brain tumors and neurodegeneration related disorders such as dementia. However, human adult brain is covered by a thick and hard skull, which has a sandwich-like structure (central spongy bone is sandwiched by compact bone on both surfaces). From the point of view of optics, skull bone is much denser than its surrounding soft tissues, resulting in more optical attenuation in the bone. From the point of view of US, there are large acoustic impedance mismatching between the boundaries of bone and soft tissues [19,20], and large acoustic attenuation in the sandwiched bone structure [21]; both greatly attenuate and distort the transcranial US. Hence, the effect of skull blocks incident light (attenuation) and FUS (attenuation and distortion) into the interior brain tissue, resulting in weaker acoustic optical modulation from human brain, comparing with the case of modulation in totally soft tissue such as abdomen or breast. On the other hand, we think that the skull effect is less in AO detection than in US/PAI, because the attenuation of optical signal in the former is less than that of US signal in the latter at the boundaries of brain/skull and skull/scalp, considering mismatching of refractive indexes and acoustic impedances among these tissues [22,23].

Recently, it has been demonstrated that AO technique has good detection sensitivity which can sense glucose concentration from 20-mm thick tissue-mimicking phantom with 26.6% accuracy in terms of mean absolute relative difference [24]. Liu et. al. studies the skull effect on AO imaging of an absorber to be attached on inner skull wall [25]. However, the setup is a transmission style, which is easier to detect AO signal than backward reflection geometry [26]. It is not suitable for *in vivo* or clinical applications. In this paper, we report a backward reflection setup of FUS-modulated light for studying the skull effect on AO sensing of brain phantom, which is useful and encourages further research in AO application *in vivo*.

## 2. Materials and methods

A laser with a single longitudinal mode and a long coherence length was used as the illuminating source (100 mW and 852-nm wavelength, CrystalLaser). The light was guided to sample surface by an optical fiber with diameters of 105 µm (mode M15L01, Thorlabs). Two commercial FUS transducers (V314-SU, 1 MHz, 25.4 mm focal length, and V382-SU, 3.5 MHz, 21.1 mm focal length) and one custom-made FUS transducer were respectively used to modulate the light in focal area in sample. The custom-made transducer has a piezoelectric disc (SP4, thickness 3 mm, CermaTec) with 50-mm diameter for generating US and a separate acoustic lens (made of aluminum material) to focus the US, which can be seen in Fig. 6(b).

**Fig. 1.**
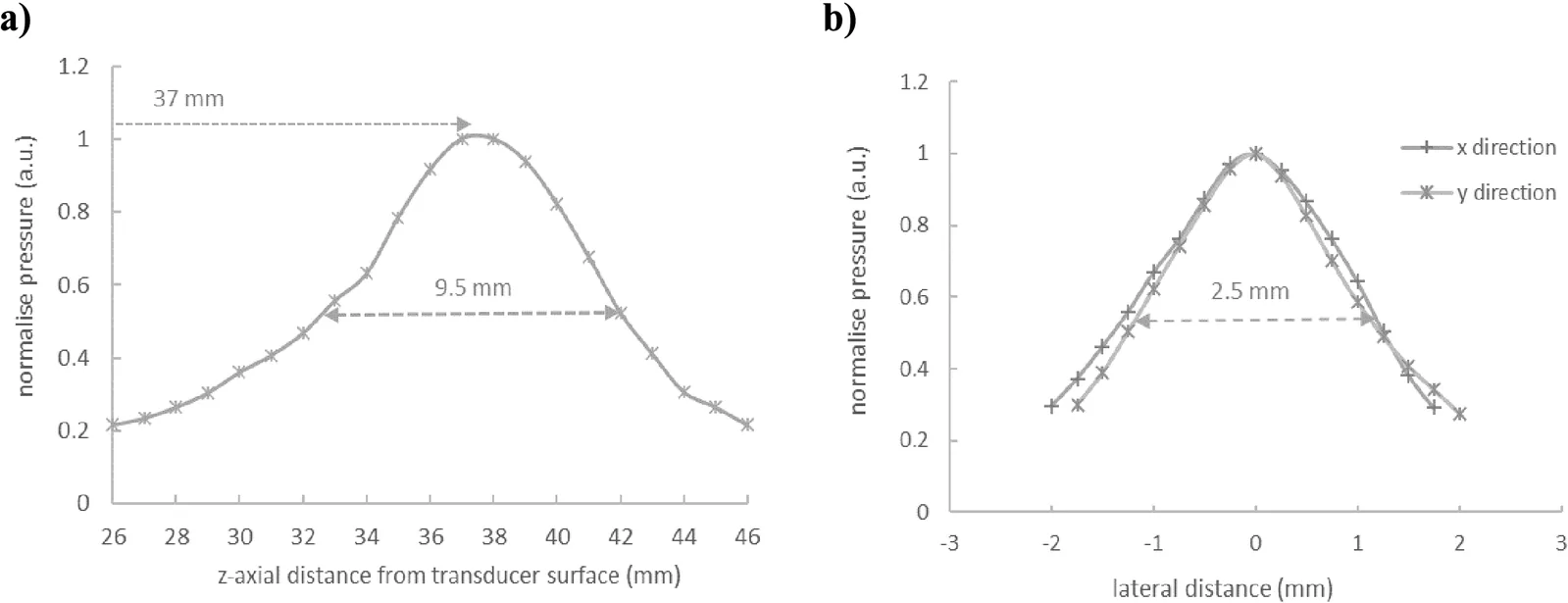
FUS pressure distribution of the self-made transducer with 0.706 MHz center frequency along z-axial direction of the transducer (a) and along lateral direction (b).

To verify the FUS pressure distribution of the custom-made transducer, a commercial hydrophone (NH1000, Precision Acoustics) was used. The hydrophone was immersed in degassing distilled water to measure US pressure produced by the self-made transducer. By scanning electrical driving frequency in sub-MHz range, the transducer generated maximal US pressure at 0.706 MHz. Hence, the self-made transducer was driven at frequency 0.706 MHz in all experiments. To measure the FUS pressure distribution for the self-made transducer, the hydrophone was assembled on a 3D translation stage, which can be moved with 1-mm step length in the axial (z) direction of the transducer and 0.25 mm step length in the horizontal (x, y) directions. Figure 1 shows the normalized pressure distribution along axial and lateral directions, in which the focal distance is 37 mm, the FWHM (full width of half maximum) focal length is 9.5 mm, and the FWHM focal width is about 2.5 mm.

**Fig. 2.**
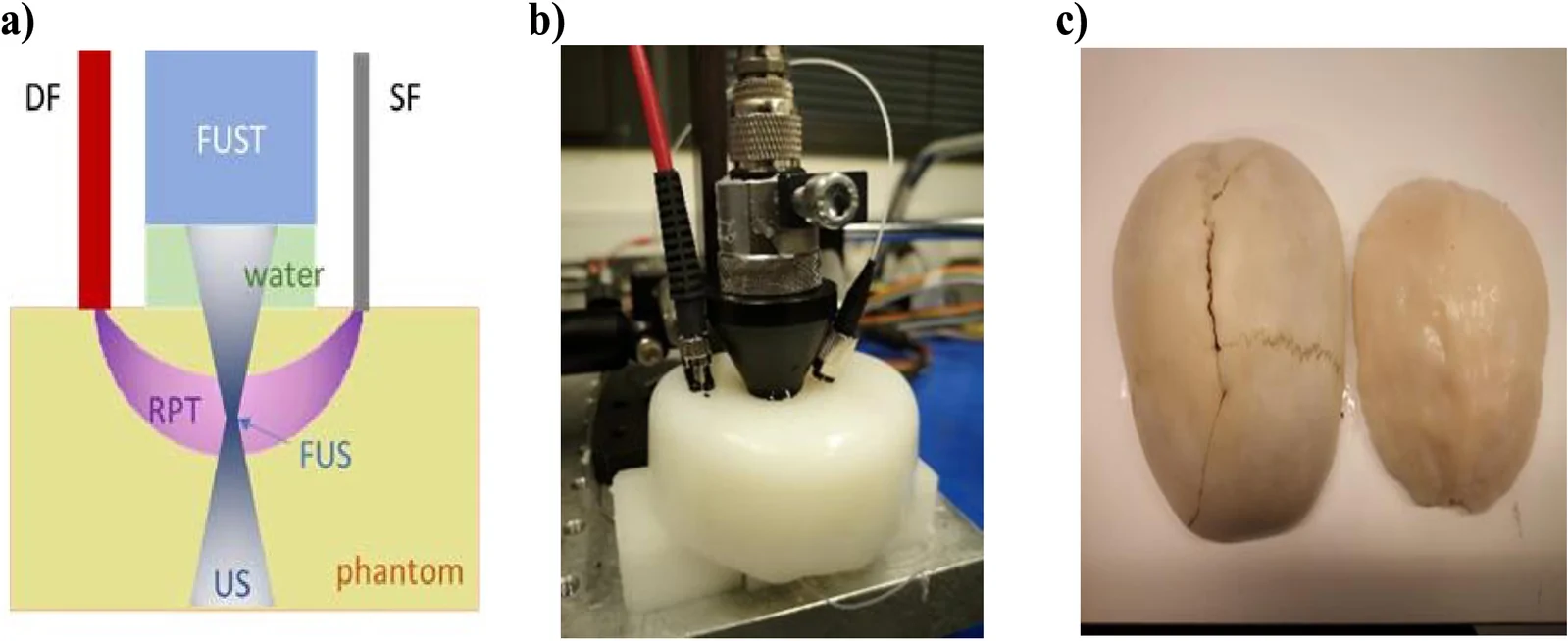
**a)** The scheme of backward sensing in this study including FUS transducer, detector fiber and source fiber pair. **b)** Corresponding measurement setup when using tissue phantom. **c)** Human adult skull specimen (left) and brain phantom (right) used in this study. DF: detection fiber, SF: source fiber, FUST: focused US transducer, FUS: focal area, and RPT: received photon trajectory.

A typical scheme of backward sensing of AO signal is shown in Fig. 2. FUS transducers were fixed in a water tank with degassing water as acoustic coupler. Tissue phantom was placed under the tank to contact water. The position of the transducer could be adjusted such that the focal region was in the tissue phantom overlapped with the banana-shape optical path between the source and detection fibers. Multi-mode optical fibers with diameters 0.2 mm, 0.4 mm, 1 mm, 1.3 mm and 2 mm were tested separately as a detector fiber at distance of 3 cm from the source fiber while the FUS transducers were placed between them. The detected light was guided to a commercial high-gain photoreceiver (OE-300-SI-30-FST, FEMTO) for signal amplification and filtering. The output from the photoreceiver was then digitized using DAQ card (PCI-5124, National Instruments) in personal computer (PC) for signal acquisition and processing by LabView software.

The soft tissue phantom was made of liquid plastic (LIQPLEXSOFT05L, Bright Baits) and Titanium Oxide Nano-powder (TiO_2_-5430MR, Nanostructured & Amorphous Materials, Inc.). This liquid plastic is identical to polyvinyl chloride-plastisol (PVCP) [27], which can be solidified after cooling down from heating. The acoustic speed and density are 1.4 mm/µs and 1.0 g/ml respectively, similar to soft tissues (∼1.5 mm/µs and 1.0 g/ml). The acoustic attenuation coefficient is $\alpha_{\text{PVCP}}(f)=(0.56\pm 0.01)f_{\text{MHz}}^{\left(1.51\pm 0.06\right)}(dB\cdot cm^{-1})$ also similar with most soft tissues in the order of MHz frequency [28]. The typical soft tissue phantom was made as below: 200-ml of liquid plastic and 600-mg of TiO_2_ powders were loaded in a glass cup (8 cm diameter) and then ultrasonically de-aggregated the powders and magnetically stirred. The stirring was continuous during the glass cup was put on an electrical oven to heat. Keeping heating twenty minutes when the temperature of the sample was up to 180 °C, then taking the magnetic stirrer away from the glass cup, switching off the oven, and letting the mixer naturally cooling down to room temperature to solidify. The solidified mixer with 8-cm diameter and 4-cm height was easy to release from the glass cup. Its optical absorption and reduced scattering coefficients are ∼ 0.02 mm^−1^ and ∼ 1 mm^−1^, respectively [27]. For making human brain phantom, the TiO_2_ powder content was increased three times in above sample making, resulting in the final reduced scattering coefficient of ∼ 3 mm^−1^ which is identical well with that of brain tissue in NIR wavelengths. This mixture was also heated to 180 °C and used to make brain phantom as described below.

A human adult skull specimen was provided for the experiments by Department of pathology in Oulu University Hospital. The skull was cleaned and totally dry, without any soft tissues on it (as shown in Fig. 2(c)). For the molding of the brain phantom the inner surface of the skull was covered by an aluminum foil and then stored in a refrigerator around 1 hour for cooling. Next, the skull was placed into cool water to decrease temperature of the liquid phantom mixture (160 °C) when the mixture was poured into the skull. After cooling the human brain phantom was solidified inside skull (see Fig. 2(c)). When taking the phantom out from the skull the aluminum foil was also removed.

## 3. Experiments and results

**Fig. 3.**
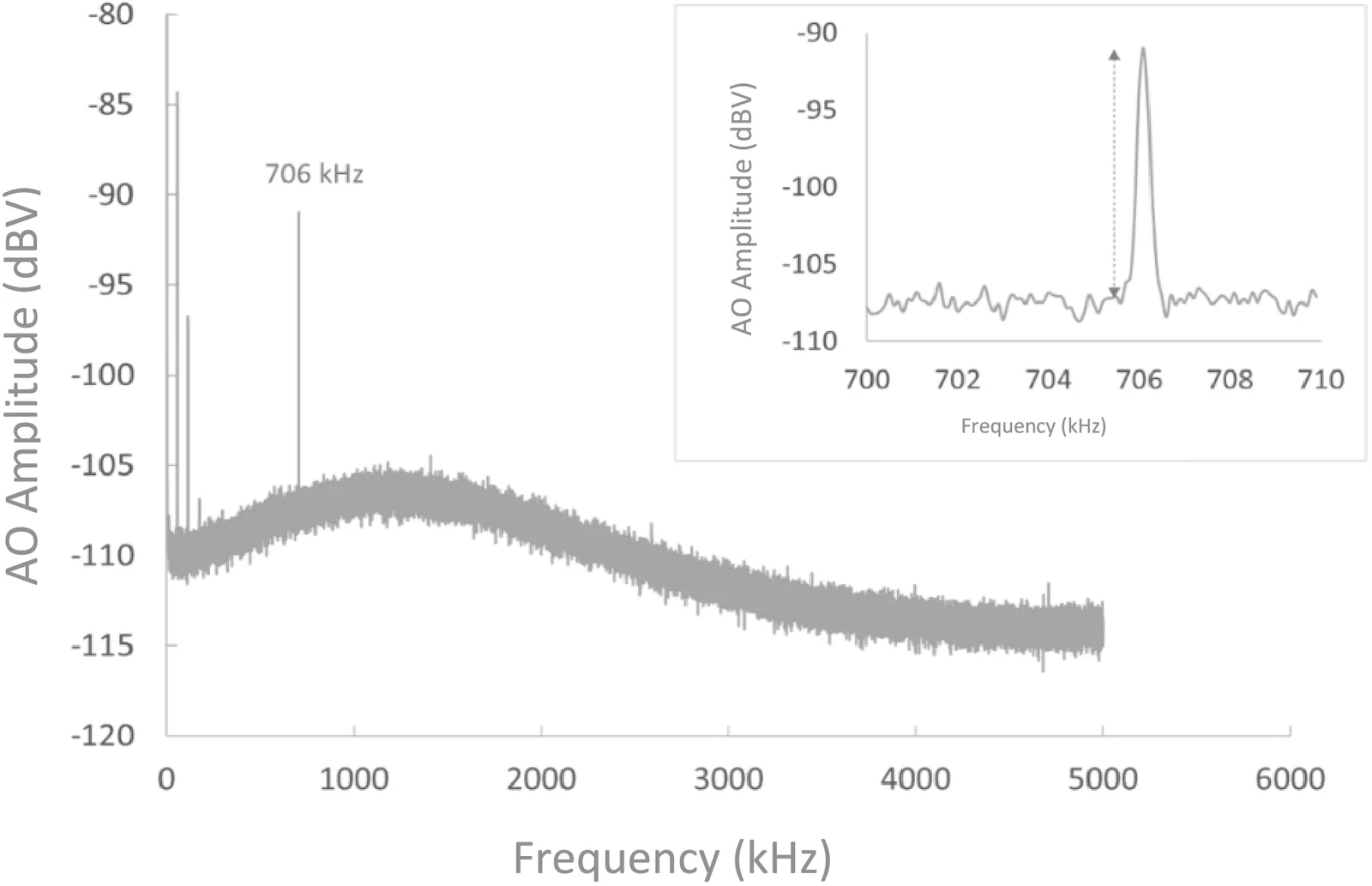
A typical AO amplitude and its enlarged part (the inserted plot) around modulation frequency of 0.706 MHz. Peaks visible at lower frequencies are probably caused by other vibrating modes of the transducer.

Because AO signal should be produced in the human brain, which is about centimeter order depth in head, the distance of source-detector fibers was kept 3 cm in this study. A typical voltage amplitude spectrum of an AO signal is shown in Fig. 3, where the self-made FUS transducer was used to modulate the incident light. The transducer was driven to generate FUS pressure 0.5 MPa, by sinusoidal voltage waves at 0.706 MHz frequency. It can be seen that the AO signal was very weak, and its power spectrum is lower than – 80 dB. To increase the signal-to-noise ratio, the power spectrum was done with 128-times averaging. As shown in the inserted plot of Fig. 3, the AO amplitude (marked as dot line arrow) at FUS modulated frequency is used as a measure of AO signal in this study. In every experiment, there were five measurements conducted to calculate the mean value and the standard deviation of the detected AO amplitude.

### 3.1. Effect of ultrasonic frequencies on AO amplitude

**Fig. 4.**
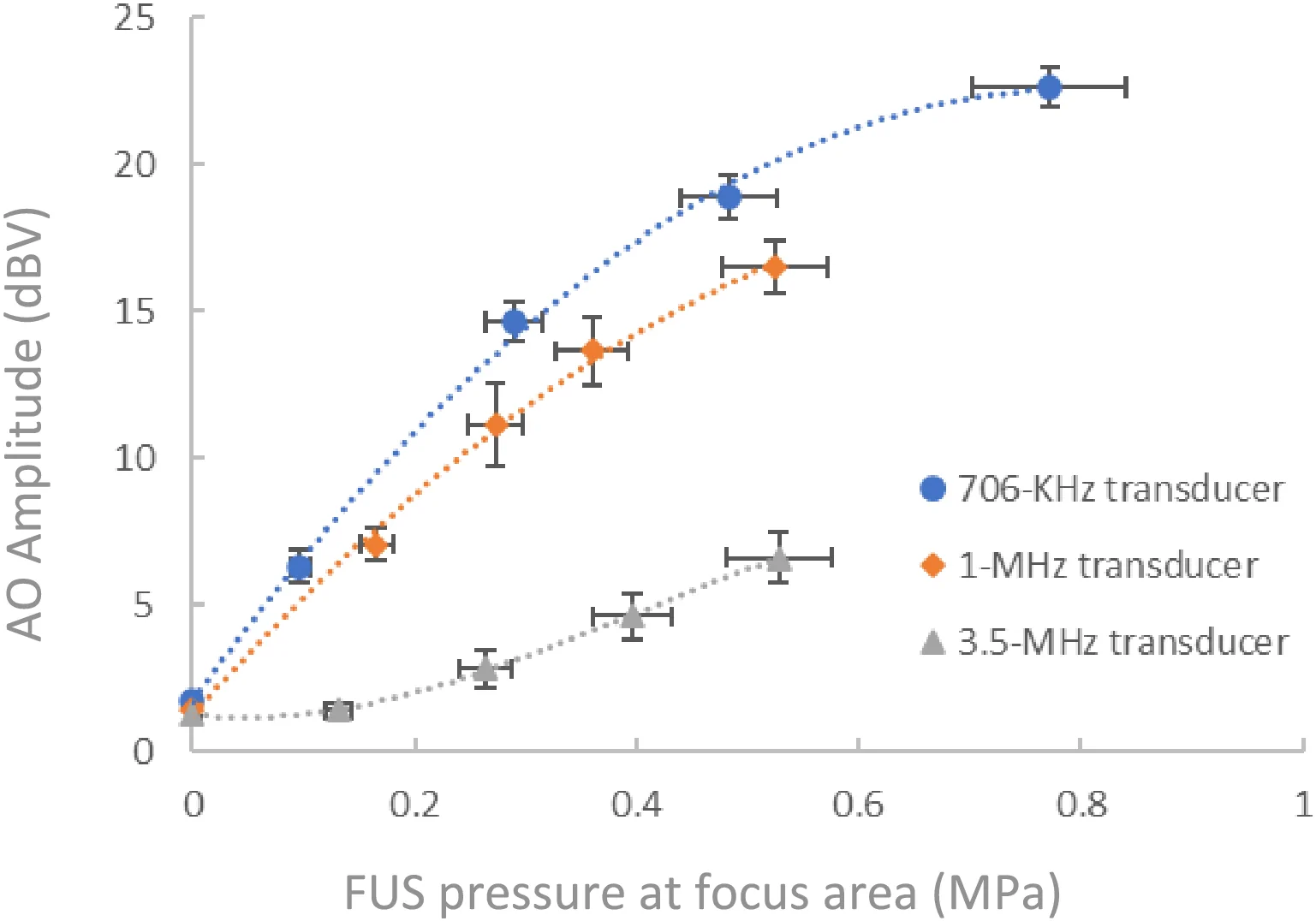
The relationship between AO amplitudeand FUS pressure to be measured by three FUS transducers with different center frequencies.

Figure 4 shows result of the variation of AO amplitude with FUS pressure emitted from three FUS transducers at their center frequencies (0.706 MHz, 1 MHz and 3.5 MHz respectively). As the FUS pressure increases, the AO amplitude is correspondingly increased, with a trend to be described by a polynomial function between 2^nd^ and 3^rd^ order. The variation trend is also consistent with simulation and experiment results published by Huang et al. [16], for example, the AO amplitude (proportional to the tagging efficiency) modulated at 1 MHz frequency is about 3 times of that at 3.5 MHz. The horizontal error bar of the pressure is due to calibration of the commercial hydrophone (18% from the product specification, which is not marked in below figures for clearing plots), whereas the vertical error bar of the AO amplitude comes from the standard deviation of five measurements. Figure 4 illustrates that lower US frequency produces higher AO signal at same FUS pressure, so the FUS transducer at lower frequency (0.706 MHz) was selected in the skull and brain phantoms study in Section 3.3.

### 3.2. Effect of fiber detection diameter on AO signal

**Fig. 5.**
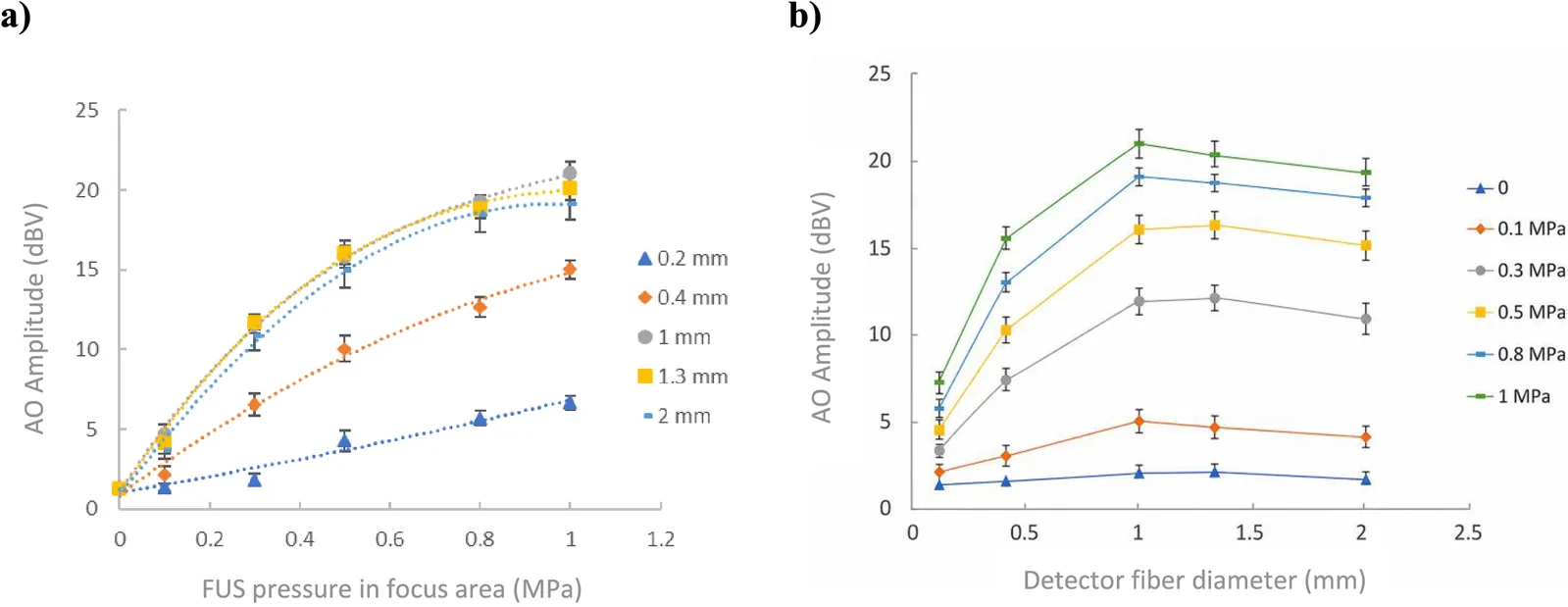
The relationship between the AO amplitude and the detector fiber diameters, under different FUS pressure. Detection fiber diameter is the parameter in **(a)** and FUS pressure is the parameter in **(b)**.

As the detection fiber received the FUS modulated light from the phantom’s surface and guided it to the photoreceiver, the fiber diameter determined the accepted light power including the tagged and untagged photons, which should relate to the modulated signal intensity. In identical experiment condition, optical fibers with different diameter were successively used to receive the modulated light from tissue phantom. We got the change of AO amplitudewith variation in the detection fiber diameter, as shown in Fig. 5, with different parameter of FUS pressure. It illustrates that the AO amplitude increases rapidly with fiber diameter in the case of small diameter, and then the AO amplitude is increased slowly to its maximum with fiber diameter in the range of 1 - 1.3 mm. When the detection fiber diameter further increased, the AO amplitude of the AO signal decreased, for example the 2 mm diameter fiber. This is possible as discussed in Section 4. The error bars in the Fig. 5 are the standard deviation of the AO amplitude under the corresponding FUS pressure.

### 3.3. Human adult skull effect on AO signal in brain phantom measurement

**Fig. 6.**
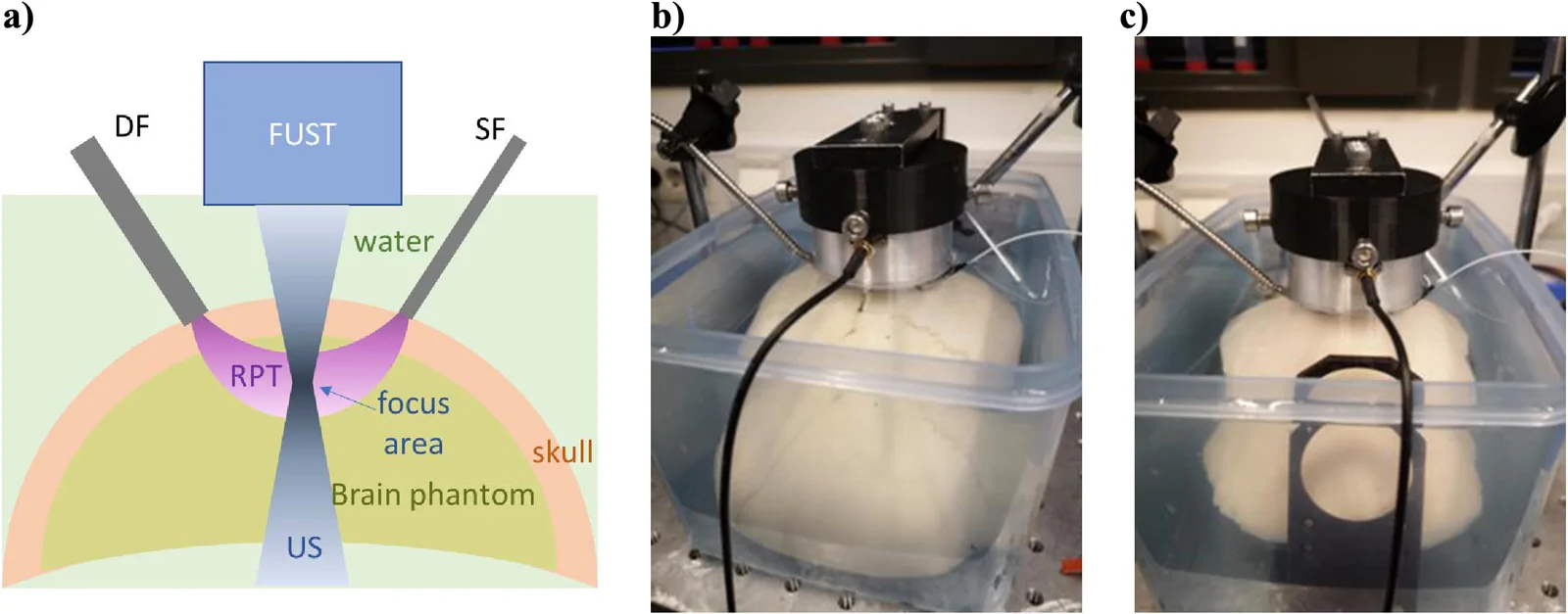
Schematic diagram of the backward-mode AO sensing experimental setup **(a)**, photograph of the experimental configuration with the adult human skull specimen placed over the brain-mimicking phantom **(b)**, and photograph of the configuration with the brain-mimicking phantom only **(c)**. DF: detection fiber, SF: source fiber, FUST: FUS transducer, RPT: received photonic trajectory**.**

Our previous study [29] showed the detected diffusively optical intensity is 10^−6^ order of incident light in the case of source-detector fiber pair with 3-cm distance on sample surface. On the other hand, the modulation depth (defined as the ratio of FUS modulated light intensity and diffusive light intensity) is in the range 10^−5^ - 10^−3^ mostly [18]. Both results confirm that AO signal has low SNR in this case. Based on the experiment results in Sections 3.1 and 3.2, we chose the 0.706 MHz FUS transducer and 1.3-mm detection fiber in this section study, which could give the largest generation and detection of AO signal in our current setups. In the experiment, both source and detection fiber axis were set a 45° angle with the surface of skull or brain phantom, as shown in Fig. 6, which could give stronger AO signal from deep tissue [30,31].

Figure 6(b) illustrates the arrangement of samples in the experiments. First, both adult skull specimen and brain phantom were immersed in degassed water, hitting the skull gently to remove air in the spongy bone between inner and outer compact bones. It should remove all air bubbles that escaped from the skull, because the air bubbles in the skull strongly attenuate and reflect transcranial US, greatly reducing FUS pressure in the brain phantom. The front end of the FUS transducer contacted with the water to achieve well acoustic coupling. The FUS transducer is located upside of the skull and kept the center focal region just under the skull (as shown in Fig. 6(a)). Here the distance between the FUS transducer and skull was 28 mm, the averaging skull thickness was 7 mm, and source-detection fiber distance was 30 mm. These dimensions kept the focal region overlapping well with photonic trajectory (pink banana shape) such that produced the most tagged photons and maximal AO signal. A similar experiment was repeated for brain phantom only, by putting the brain phantom on the skull position before. Each experiment arranges were shown in Fig. 6(b). A black metal part was used to keep the brain phantom from moving.

**Fig. 7.**
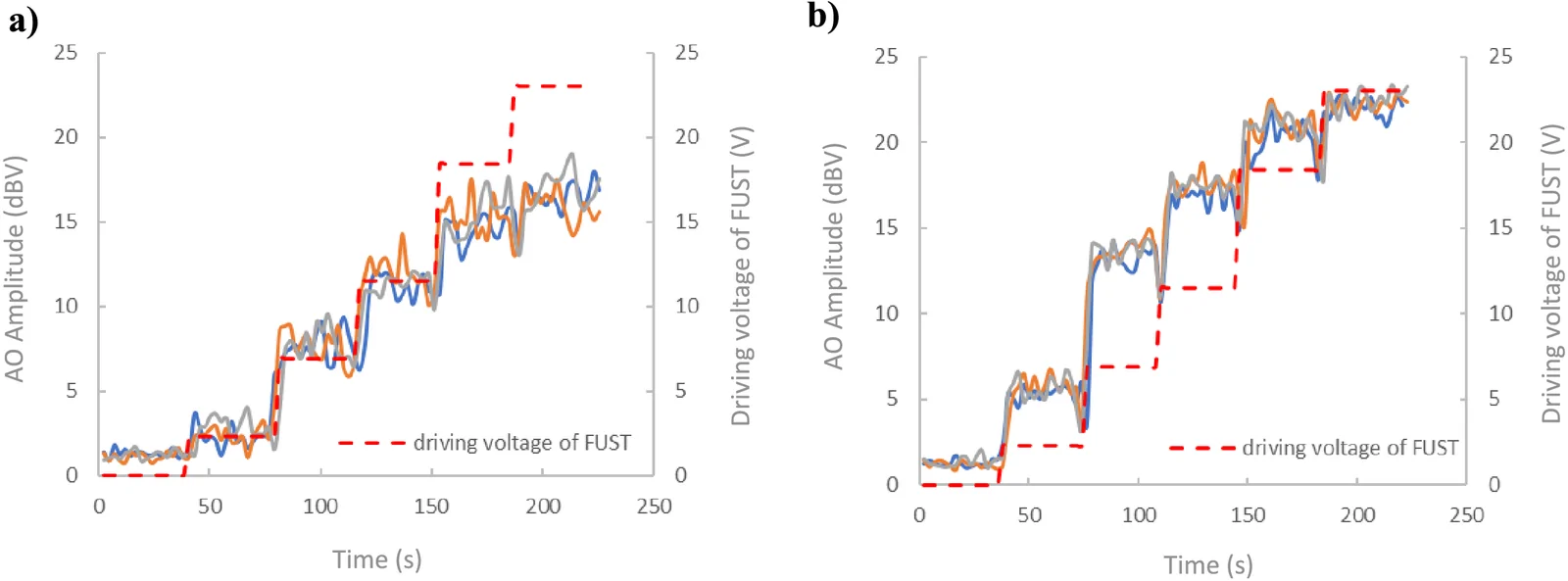
Recorded AO amplitude variation with time in the experiments of skull/brain phantom **(a)** and brain phantom only **(b)**, when driving voltage of the FUS transducer increased. Three solid curves in every plot show repeating three times in every experiment. FUST stands for FUS transducer.

Figure 7 shows the variation of the AO amplitude with time when the driving voltage of FUS transducer is stepped up in definite time (∼ 40 s) during the experiments; three solid lines in the graphs record three measurements where the dot line marks the change of the driving voltage of FUS transducer with time. There is a small level of AO amplitude signal when FUS driving voltage is zero, which comes from the recorded noise floor (similar with the floor in the inserted plot in Fig. 3). The recorded AO amplitude has a small drop just after the driving voltage increasing, and then rises in a few seconds and trends to stable with a finite shift.

**Fig. 8.**
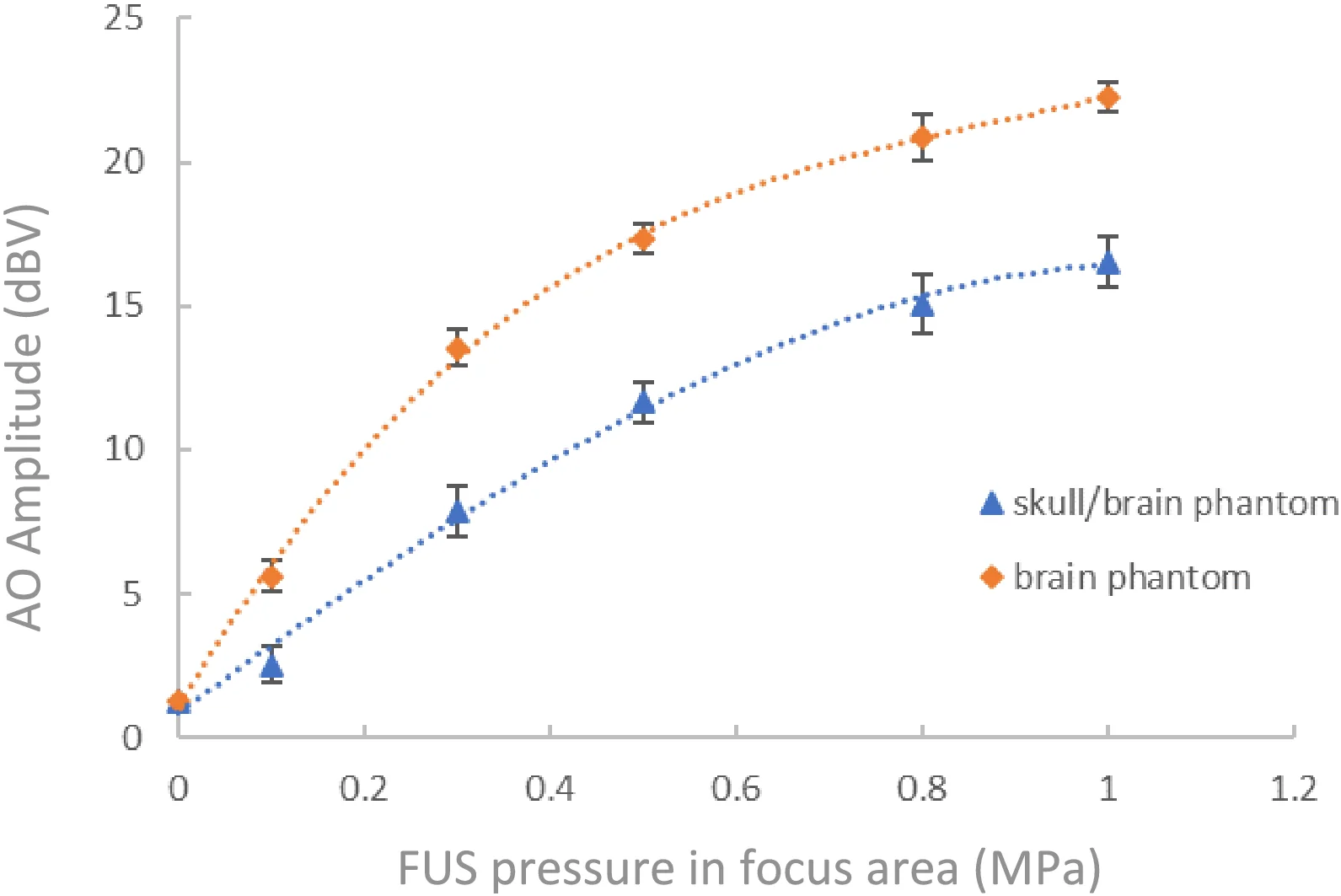
Relationship between AO signal amplitude and FUS pressure in both phantoms. The solid marks are average values, and the error bars are standard deviation in three repeating measurements.

A short transient is observed immediately after each increase in the driving voltage. This transient is attributed to the response of the measurement system and the signal processing used to estimate the AO amplitude, rather than to the establishment of the ultrasonic field itself. The averaged values and standard deviations of AO amplitude following the FUS pressure increase are illustrated in Fig. 8, which are extracted from data in Fig. 7 after the FUS fields to be stable in every pressure step-up. The precent decrease of the AO amplitude when the brain phantom is covered by the skull (skull effect) is listed in the right column of Table 1; for example, the AO amplitude is reduced to 49% by the skull when the FUS pressure is 0.5 MPa. The error at FUS pressure 0.1 MPa is apparently larger than those at higher pressures, because of floor level of AO amplitude at absence of FUS pressure (the floor level is 1.28 dB at 0 MPa in Fig. 7 and Fig. 8).

**Table 1.** The effect of skull on AO amplitude produced in the brain phantom

FUS pressure (MPa)	Brain phantom (dBV)	Skull/brain phantom (dBV)	Effect of skull (dB)
0.1	5.61	2.54	-3.07
0.3	13.54	7.84	-5.7
0.5	17.34	11.65	-5.69
0.8	20.83	15.03	-5.8
1	22.26	16.52	-5.74

## 4. Discussion

There are several detection techniques in FUS modulated light to be developed, but their clinical application is limited because of their drawbacks including 1) low SNR for single-element intensity detection 2) long detection time for parallel lock-in detection, 3) slow response time for photorefractive crystal-based interferometry, and 4) bulky and expensively cryogenic instrument for spectral-hole burning in rare-earth crystal [9,10]. Moreover, the clinical application usually requires backward detection which optical emitter and detector locates on same side of human body. But the backward detection has lower sensitivity than forward detection for AO signal [26]. In this study, thanking the high sensitivity of the photoreceiver, our setup has a good sensitivity to detect AO signal in brain phantom with a distance of source-detection fiber pair up to 3 cm. The achieved AO amplitude is ∼ 25 dB in the brain phantom at depth of 12 ∼ 15 mm when using 128 times of signal averaging. Although the presented setup is based on single-element detection technique, it uses optical fiber pair and has fast signal processing, which is beneficial to combine other clinical techniques.

FUS pressure and frequency closely relate to modulation efficiency. US is a mechanical wave; its pressure causes the particles or cells movement in tissues. For the same FUS pressure, higher US frequency corresponds to lower AO modulation. This is because particle displacement is inversely proportional to the US frequency. Thus, higher frequency US generate smaller particle displacements for the same pressure, resulting in lower tagging efficiency and modulation amplitude. On the other hand, high FUS pressure together with low frequency has more opportunity to generate longer movement and larger changes of the index of refraction in the tissues. We measured the AO amplitude at the US frequency, which is produced by fundamental harmonic. However, there are tagged photons with higher orders of US frequency [17]. As FUS pressure increases, the non-linear effect or the number of higher orders tagged photons is probably increased (similar the case of multi-photon absorption in nonlinear optics), whereas the number of the first order tagged photons trends to unchanged. Therefore, the AO amplitude produced by the first order tagged photons is gradually saturated with the increase of FUS pressure (as shown in Fig. 4).

Figure 5 shows that the AO amplitude increases fast with detection fiber diameter in the case of small diameter, and then increases slowly to the maximum as fiber diameter increases to 1 - 1.3 mm. It is understood that the AO signal increases with fiber diameter, because large fiber diameter has a large optical etendue or acceptance. However, as the fiber diameter further increases to 1 mm or more, the AO amplitude AO amplitude gradually saturates and no longer increase. This is due to the AO signal produced by speckle pattern which is produced by the interference of the tagged photons following random pathlength difference in tissue phantom. The speckle pattern consists of a distribution of random bright and dark grains. Therefore, the phases of the time-varying intensity modulation of these speckle grains are random. If detection fiber diameter is large enough that the modulated speckle grains in it have enough large of phase difference, the averaging bright of the speckle pattern in the fiber diameter does not increase anymore or even decrease. Therefore, the AO signal gradually saturates and does not increase with detection fiber diameter. This result is supported by Leveque-Fort’ simulation [32], which single element detector cannot directly increase the strength of AO signal simply by increasing the detector’ area.

The human adult skull reduces the AO signal, as shown in Fig. 8, where the AO amplitude is reduced when the brain phantom is covered by the skull specimen. The observed reduction, approximately 5.7 dB for FUS pressures between 0.3 MPa and 1 MPa (Table 1), is consistent with the expected combined influence of several physical mechanisms introduced by the skull, as discussed below.

Acoustic attenuation is the dominant contributing factor. Using the measured average skull thickness of 7 mm and the published attenuation coefficient of 13.3 dB/cm at 1 MHz reported by Pinton et al. [21], the estimated one-way pressure attenuation is approximately 9.3 dB at 1 MHz, or approximately 6.6 dB at our operating frequency of 0.706 MHz assuming linear frequency scaling. This corresponds to a pressure transmission of approximately 47%, and is in good agreement with our measured AO amplitude reduction of ∼5.7 dB. This estimate is also consistent with the acoustic transmission measurements across parietal bone reported by Riis et al. [33]. Beyond acoustic attenuation, several additional mechanisms may contribute to the observed AO signal reduction. First, the speed-of-sound mismatch between skull bone (∼2,500–3,000 m/s in cortical bone) and the surrounding water and soft tissue (∼1,500 m/s) introduces phase aberrations that can axially shift and spatially broaden the acoustic focus [34]. In our experimental geometry, with the transducer placed 28 mm above the skull surface and a skull thickness of ∼7 mm, a non-negligible focal shift is expected, which may reduce the spatial overlap between the acoustic focus and the banana-shaped photonic trajectory, thereby reducing the number of tagged photons beyond the reduction expected from acoustic attenuation alone. Second, the skull bone has substantially higher optical scattering and absorption than soft tissue in the NIR wavelength range, which reduces the fluence of both incident and backscattered photons traversing the skull, contributing to a reduction in the detected AO amplitude [29]. Third, despite the degassing procedure described above, residual air pockets within the diploe layer of the skull cannot be entirely excluded; even small air inclusions are known to cause strong acoustic reflection and scattering, introducing additional variability in the transmitted acoustic pressure. Finally, the heterogeneous microstructure of the skull may induce speckle decorrelation at the detector, slightly reducing the detected modulation depth. While acoustic attenuation is consistent with the primary observed signal reduction, these additional factors likely contribute to the remaining variability across measurements and should be considered in the design of future transcranial AO studies.

AO modulated signal is less affected by the skull than transcranial US, since the FUS traverses the skull only once in the AO configuration, compared with twice (outward and return paths) in pulse-echo transcranial US. On the other hand, in the case of transcranial photoacoustic detection, photoacoustic waves were generated by optical absorption substance in brain, with a form of nanosecond pulse US in couple of MHz frequencies. Although photoacoustic waves propagate out from the skull wall one time, the pulsed waves with a few MHz frequencies propagate in 360° from the brain are easier to be distorted and attenuated, comparing with directional, continuous, or burst US in the case of AO modulation. Hence, in the context of transcranial brain sensing and imaging, AO modulation offers a distinct advantage in terms of resilience to skull-induced signal degradation.

However, the progress of AO sensing or imaging is slower than its competitors (optical and PA) in periclinal and clinal application. The main drawback of current AO techniques is still low SNR, i.e., weakly modulated signal is submerged in huge unmodulated background light. Although Huang et al [17] declared the tagged efficiency could be up to 70% totally (or <10% for 1^st^ order modulation), they only considered the tagged photons and untagged photons in FUS region and did not consider huge untagged photons outside of the FUS region which is popular case in detecting diffusive light from deep tissue. This greatly overestimates the modulation efficiency in deep tissue sensing or imaging, their result therefore is only suitable for the case of superficial tissues. In the case of deep tissues, the AO modulation efficiency is much smaller, as Wang’s simulation result [18]. To increase the modulation depth or SNR, parallel detection by an optical fiber bundle together with a multiple element array is a good issue, although it adds the complexity of measure system and probably delays signal processing time in some degrees. Anyway, more advanced techniques need to be developed before transcranial application of human brain in vivo.

## 5. Conclusions

FUS modulated light sensing of brain is inevitably affected by skull, because of mismatching in refractive index and acoustic impedance between skull and soft brain tissues. Encouragingly, our experiment result shows that the AO amplitude only reduces about a half when the signal to be produced in brain phantom goes through an adult skull specimen at 0.5 MPa FUS pressure. This moderate effect of the skull encourages transcranial AO study of brain, comparing with great effect of the skull on transcranial US or PA. Our backward sensing of FUS modulated light setup based on a source-detection fiber pair, which is convenient and prepared for next *in vivo* or pre-clinical measurement. Meanwhile, we studied the effect of FUS frequency on AO signal and showed sub-MHz FUS more efficient than that with higher frequency. The detection fiber was also optimized for its diameter in the case of experimentally used photoreceiver, maximal AO signal was received by the fiber with diameter 1 ∼ 1.3 mm.

## Data Availability

Data underlying the results presented in this paper are not publicly available at this time but may be obtained from the authors upon reasonable request.
